# Guideline concordant opioid therapy in Veterans receiving VA and community care

**DOI:** 10.1186/s12913-024-11742-1

**Published:** 2024-10-26

**Authors:** Phillip Ma, Yan Cheng, Joseph L. Goulet, Friedhelm Sandbrink, Cynthia Brandt, Chris Spevak, Jacob T. Kean, William Becker, Alexander Libin, Nawar Shara, Helen M Sheriff, James S. Houston, Jorie Butler, Elizabeth T. Workman, Rajeev M Agrawal, Joel Kupersmith, Qing Zeng-Treitler

**Affiliations:** 1 Washington DC VA Medical Center, Washington, DC USA; 2 George Washington University, Washington, DC USA; 3 Yale University, New Haven, CT USA; 4 Georgetown University School of Medicine, Washington, DC USA; 5 MedStar Health, Columbia, MD USA; 6 The University of Utah, Salt Lake City, UT USA; 7 Georgetown Howard Universities Center for Clinical and Translational Science, Washington, DC USA; 8 VA Connecticut Healthcare System, West Haven, CT USA; 9 University Biomedical Informatics Center, 2600 Virginia Ave NW, Suite 300, 20037 Washington, DC USA

**Keywords:** Opioid therapy disorder, Concordant care, Drug adherence, Mono vs dual-system

## Abstract

Guideline concordant opioid therapy is a key part of the concerted effort to address the opioid crisis in the United States. The study aimed to compare the rates of guideline concordant care between veterans who solely used VA services (mono users) and veterans who used both VA services and community care (dual-system users). We used electronic health record data from the Washington DC and Baltimore VA Medical Centers from 2015 to 2019. We provided descriptive statistics as well as generalized estimating equations models to find associations between mono vs. dual-system users and each guideline outcome, controlling for demographic factors and comorbid conditions. The study found that overall rates of guideline concordant care were high in both mono and dual-system users with over 90% adherence rates for the majority of recommendations. However, there were variations in adherence to specific guidelines, with urine drug screening at initiation being the least commonly followed recommendation (8.9% of mono-user opioid initiators and 11.2% of dual-user initiators). This study also found that there was no consistent pattern of higher guideline adherence in mono vs. dual-system users but did show that through the course of this study (2015–2019) overall rates of guideline concordance increased. Future research will explore additional guideline recommendations and potential coordination issues among dual-system users.

**Supplementary Information:**

The online version contains supplementary material available at 10.1186/s12913-024-11742-1.

## Introduction

The United States is in the middle of an overdose crisis that began with the increasing use of prescription opioids since the mid-1990s [1–5]. Subsequently, the increasing use of illicit substances including heroin and fentanyl, and the use of psychostimulants have further worsened the overdose crisis in the United States [6]. According to the Centers for Disease Control and Prevention (CDC), over half of a million people died from overdoses involving opioids between 1999 and 2020 [7]. 

In response to the crisis, clinical practice guidelines based on the available scientific evidence, were developed and published, including several by the CDC and by the Department of Veteran Affairs (VA) together with the Department of Defense (DOD) [8–20]. These guidelines provide guidance for clinicians to improve pain management and patient safety while acknowledging that clinicians need to make decisions according to the needs of individual patients and available resources. While deviation from guideline recommendations is warranted in some cases, providing guideline concordant care is an important quality improvement goal in the management of opioid therapy [21]. 

The Veteran Health Administration is the largest integrated healthcare system in the US, serving 9 million enrolled Veterans each year. From 2012 to 2020, VA’s Opioid Safety Initiative reduced the prescription opioid use in Veterans by 64%. The reductions in the numbers of patients receiving opioids and benzodiazepines concurrently, on long-term opioids, or very high doses of opioids were even greater, ranging from 70 to 87% [22]. 

Meanwhile, care coordination remains a challenge in the management of chronic pain and opioid therapy. Many VA patients receive care from community providers. About 80% of the Veterans have private health insurance. Through the Veterans Choice Program (VCP) and the Veterans Community Care Program (VCCP), Veterans could access community care paid for by the VA. For this study, we analyzed Veterans receiving care between 2015 and 2019, i.e., during the VCP and then later in the renamed VCCP. Prior studies have examined the receipt of opioid prescriptions, diagnosis of Opioid Use Disorder (OUD), and risky opioid therapy in Veterans receiving VA and non-VA care, using data from the Medicare and health information exchange [23, 24]. The Guideline discordant opioid therapy in Veterans who use VA service and VA paid community care (VCP/VCCP), however, has not been analyzed.

This study is part of a larger project focusing on care coordination and opioid therapy. In the project, we have already assembled a cohort of patients comprised of mono users (active VA patients who did not use VCP/VCCP services) and dual-system users (active VA patients who also used VCP/VCCP services), using the 2015 to 2019 electronic health record (EHR) from Washington DC and Baltimore VA Medical Centers [25]. Our prior analyses of the cohort suggest that dual-system users are at a higher risk of opioid initiation, continued use, and misuse [25]. In this study, we measured and compared the guideline discordant opioid therapy in the mono and dual-system users.

## Methods

### Study population and data sources

**Fig. 1:**
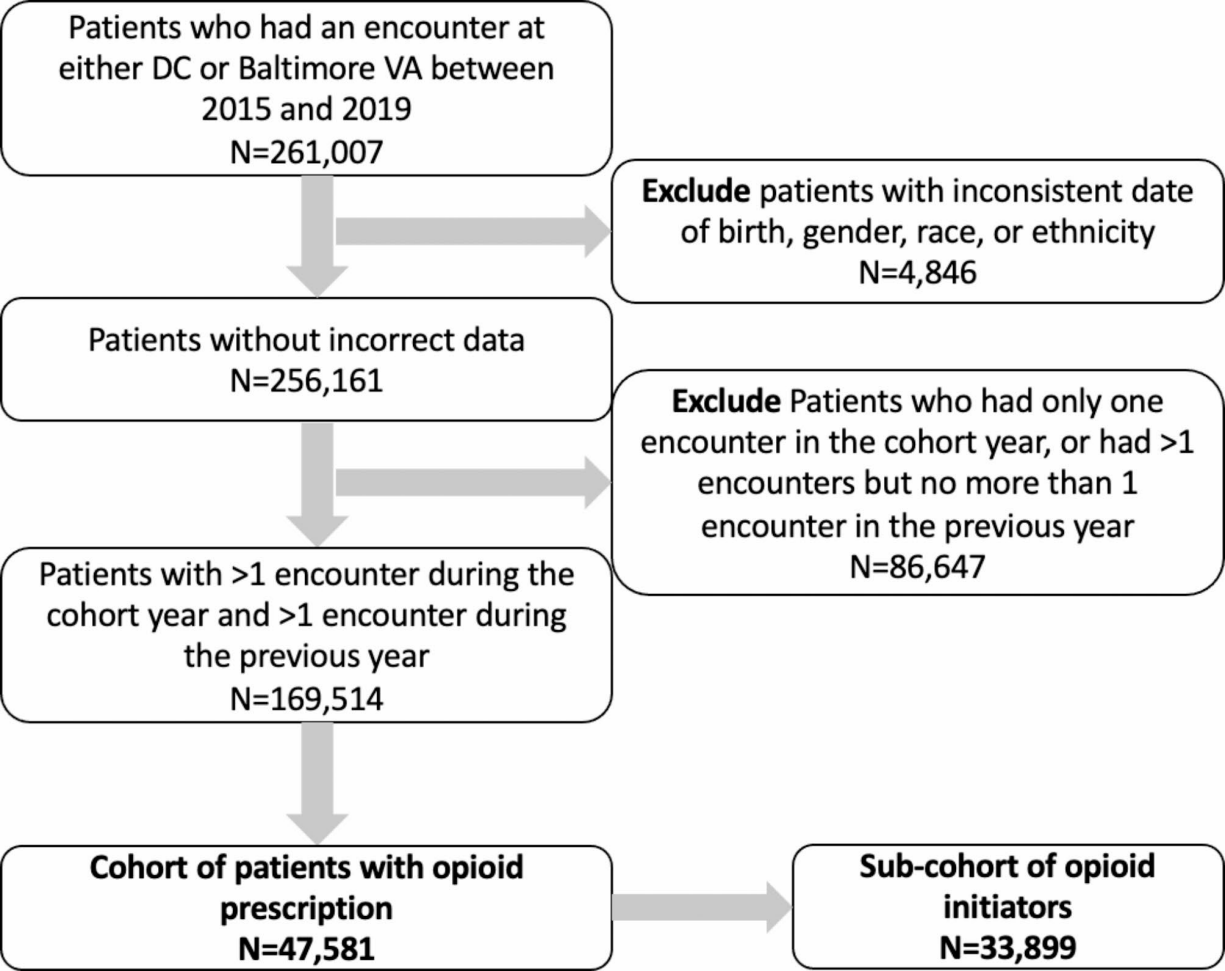
Cohort selection flow chart

We used a previously assembled cohort (*n* = 169,514) consisting of active patients from 2015 to 2019 who had at least two encounters from two consecutive calendar years in the Washington DC or Baltimore VA Medical center. (VA IRBnet protocol #1607134) Because patients’ use of VCP/VCCP changes over time, we treated each year as a separate cohort. Baseline demographic and clinical characteristics were extracted from the VHA electronic clinical and administrative data sources in the Corporate Data Warehouse (CDW) before the January 1st of each year (index date). We then limited the cohort (Fig. 1) to those with opioid prescription (*n* = 47,581 unique users, 88,785 calendar year users) and sub-cohort to opioid initiators (*n* = 33,899 unique initiators, 40,032 calendar year initiators).

### Study exposure

The main exposure of the study is the VCP/VCCP use, which was identified using a VA stop code designated for the community care program. A patient without any VCP/VCCP encounter in a calendar year is defined as “mono” user and a patient with a VCP/VCCP encounter is classified as a dual-system user for that year.

### Study outcomes

Based on the CDC and VA/DOD guidelines, our study included the following outcome measures (Table 1).

**Table 1:** Functional definitions of the guideline recommended practice

**Variable**	**Functional Definition**
**Initiate opioids with immediate release**	Initiation was defined as the first opioid prescription in the VA electronic health record (EHR) database, after a one-year period without any opioid treatment. The condition is satisfied if the dispensed opioid was an immediate release formulation.
**Re-evaluate between 1-4 weeks of initiation**	For each patient with an opioid initiation, outpatient visits were gathered. This condition is satisfied if the patient had an outpatient visit within 28 days of their opioid initiation.
**Urine drug screening at initiation**	For each patient with an opioid initiation, lab results matching urine drug screenings were gathered. This condition is satisfied if there is one such occurrence of a urine drug screening within 30 days of their opioid initiation.
**Re-evaluate every 3 months**	For each patient with an opioid prescription, the total length of treatment was calculated using the days supplied, reported in the prescription. Then for each patient, the total number of outpatient visits was counted within the treatment window. This condition is satisfied if the patient had one appointment for every 90 days of opioid treatment.
**Avoid more than 89 morphine milligram equivalents/day**	For each patient with an opioid prescription, the total Morphine Milligram Equivalent per day was calculated by converting the dosage per day contained in the prescription to the equivalent MME. The condition is satisfied if the MME/day for a patient is less or equal to 89.
**Avoid concurrent prescription of Opioids and Benzodiazepines**	For each patient, treatment windows (extrapolating forward from Fill Date using Days Supplied) for opioids and any benzodiazepines were retrieved. The condition is satisfied if there was no overlap between treatment windows between classes of medication.

### Covariates

Covariates including demographic and clinical variables were derived from VA data on the index date. We also identified selected comorbid diagnoses recorded before the index date of any given cohort year. The definition of comorbid diagnoses was defined using the criteria established by the VA MSD cohort [26] and derived from the ICD9/10 codes. Age was measured as a continuous variable and the other covariates were measured as categorical variables.

### Statistical analyses

We conducted analyses by year, accounting for the repeated observations on patient and intra-class correlations within patients. We first summarized the baseline characteristics of the dual-system and mono users for the two cohorts: opioid users and opioid initiators, respectively. We then compared the outcomes between the dual-system and mono user groups.

The main independent variable was the care pattern: dual-system user group vs. mono group. We conducted Chi-square and Student’s *t*-test to compare patient demographics and comorbid conditions between dual-system users vs. mono users for each cohort. We set an alpha level for significance at 0.05. There outcomes including ‘initiate opioids with immediate release’, ‘re-evaluate between 1–4 weeks of initiation’ and ‘urine drug screening at initiation’ were compared between the dual-system and mono user groups in the opioid initiators; three outcomes including ‘re-evaluate every 3 months’, ‘avoid more than 89 morphine milligram equivalents/day’, and ‘avoid concurrent prescription of opioids and benzodiazepines’ were compared between the dual-system and mono user groups in the opioid users. Due to the large sample sizes of both cohorts, a very small difference between groups may be statistically significant. Therefore, we also calculated absolute standardized difference (ASD) for each comparison besides reporting value of *p*, for which ASD > 10% indicates imbalanced characteristics between two groups. We further conducted generalized estimating equations (GEE) models for four outcomes except for ‘initiate opioids with immediate release’ and ‘re-evaluate every 3 months’, because the rates of two outcomes were > 99%, for which there was insufficient variety to build a model. GEE models are useful for analyzing longitudinal and clustered data, and have an advantage in estimating unbiased population-averaged parameters and their standard errors, even in cases of possible mis-specification of the correlation structure [27]. We checked correlation structure of each model: if all observations over time had the same correlation then we set within-subject covariance as exchangeable and if correlation between all timepoints was different, then we set it as unstructured. The GEE models were fitted to estimate odds ratio and relative risk of each of the outcomes between two groups (dual users vs mono users) with or without adjustment for other covariates. All analyses were conducted using SAS 9.4. (Cary, NC).

## Results

**Fig. 2:**
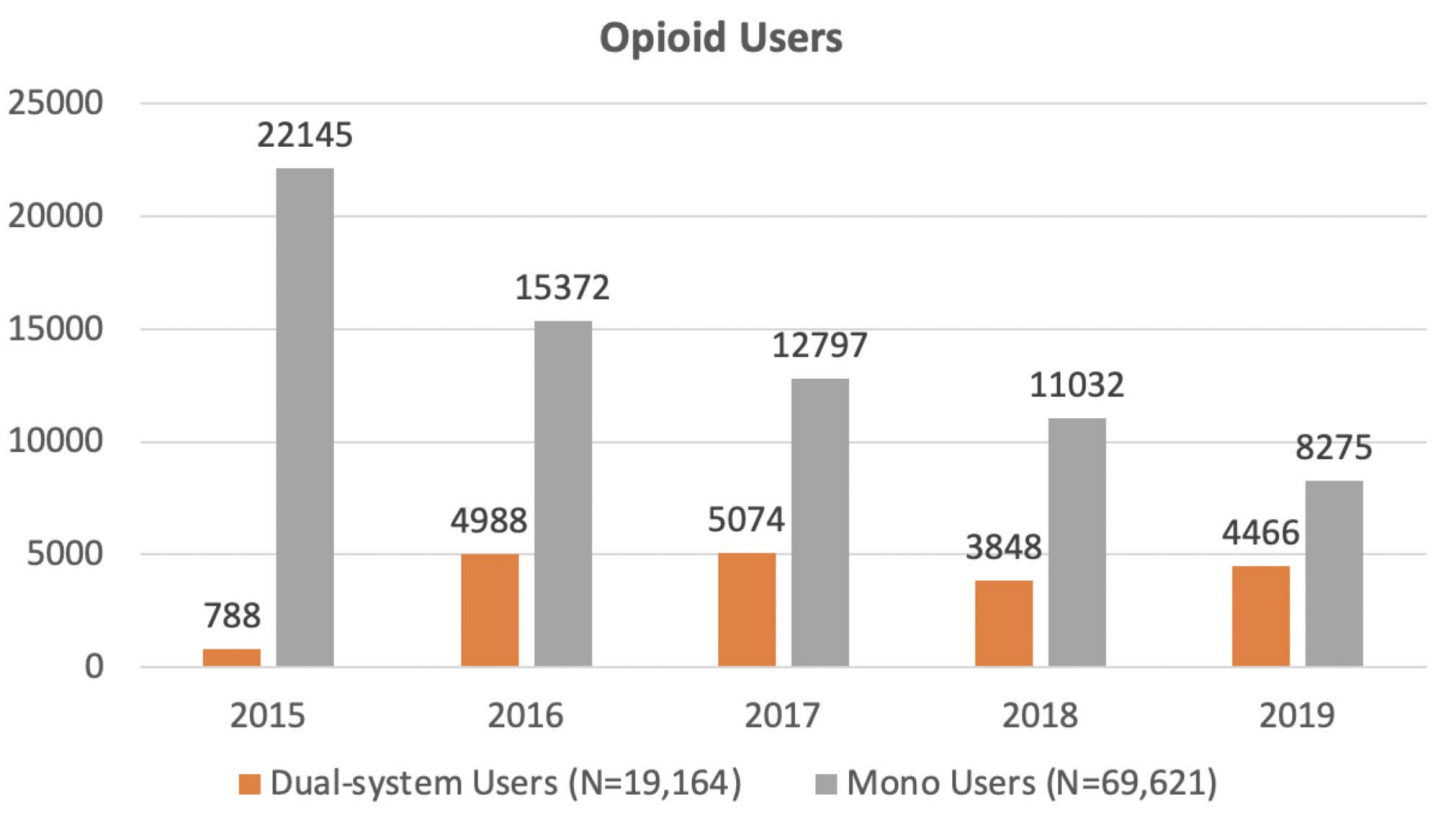
Numbers of opioid users who are mono or dual-system users from 2015 to 2019

**Fig. 3:**
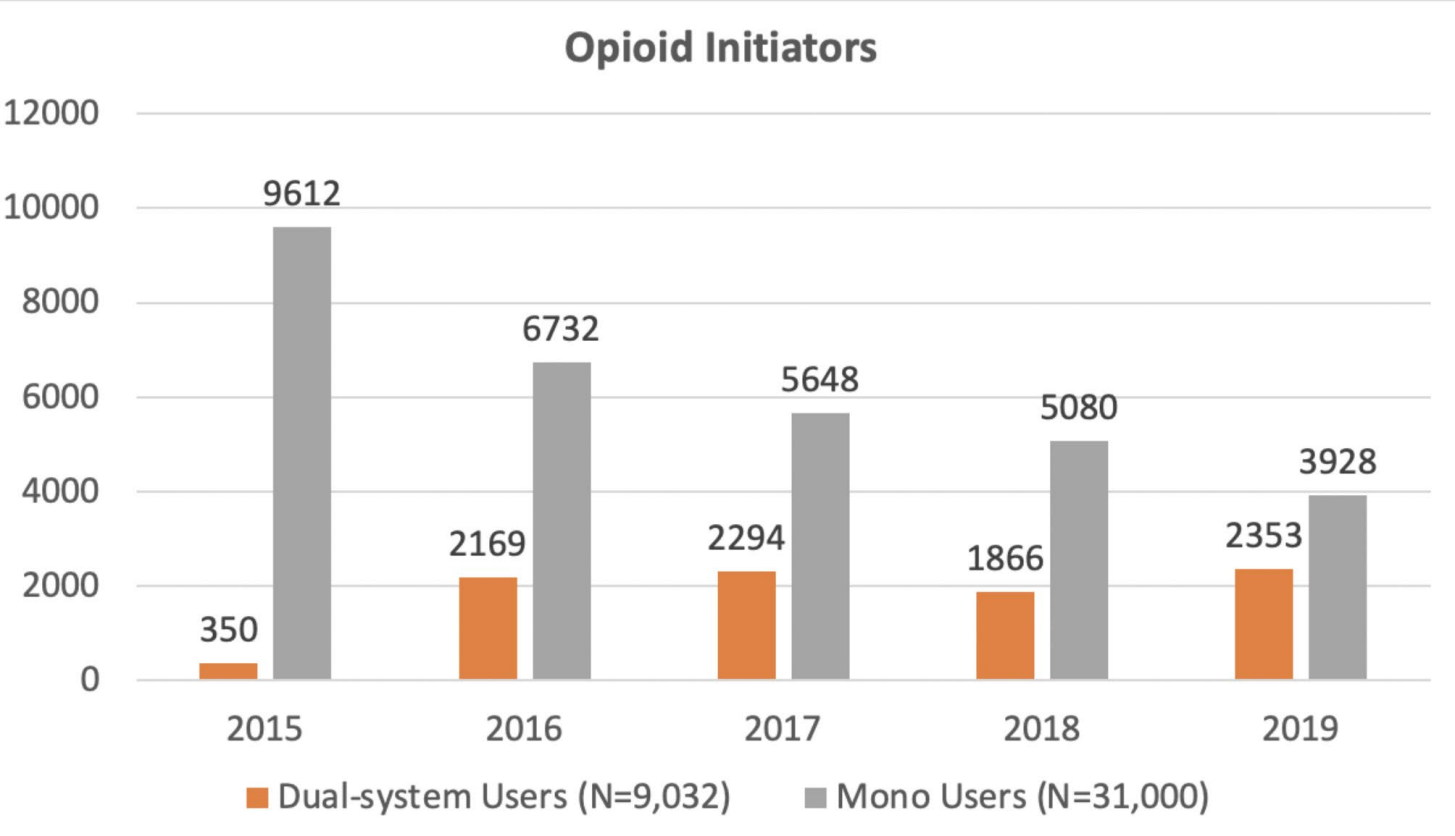
Numbers of opioid initiators who are mono or dual-system users from 2015 to 2019

From 2015 to 2019, the number of mono users who are opioid users and initiators has declined steadily. As the VCP/VCCP program became more widely utilized, the number of dual-system users who are opioid users (Fig. 2) and initiators (Fig. 3) saw a big increase from 2015 to 2016, and then remained relatively stable.

Among the opioid users, the characteristics of the dual-system users differ from those of the mono users in age, gender, race, and a few comorbid conditions. (Table 2) Compared to Mono users, dual users were younger (59.1 vs. 63.0 years), had higher proportion of females (21.8% vs. 10.8%) and Black (55.2% vs. 44.7%), and higher prevalence of comorbid conditions (PTSD: 35.9% vs. 29.5%, anxiety: 43.1% vs. 36.7%, depression: 59.5% vs. 51.7%, neck pain: 57.2% vs. 49.4%, and back pain: 76.4% vs. 72.3%). The same differences can be observed in opioid initiators (Table 3).

**Table 2:** Baseline characteristics of the opioid user cohort

	**Dual users (** ***N*** **=19164)**		**Mono users (** ***N*** **=69621)**		***p*** **-value**	**ASD (%)**
	**Mean/N**	**Std/%**	**Mean/N**	**Std/%**		
**Age**	59.1	13.6	63.0	14.1	<0.0001	**15**
**Gender**					<0.0001	
**Female**	4175	21.8%	7496	10.8%		**30**
**Male**	14989	78.2%	62125	89.2%		**30**
**Race**					<0.0001	
**White**	7367	38.4%	34623	49.7%		**23**
**Black**	10586	55.2%	31120	44.7%		**21**
**Others**	381	2.0%	1099	1.6%		3
**Unknown**	830	4.3%	2779	4.0%		2
**Ethnicity**					<0.0001	
**Non-Hispanics**	18157	94.7%	66321	95.3%		2
**Hispanics**	540	2.8%	1345	1.9%		6
**Unknown**	467	2.4%	1955	2.8%		2
**Comorbidity**						
**Hypertension**	13478	70.3%	53204	76.4%	<0.0001	**14**
**Diabetes Mellitus **	7296	38.1%	27865	40.0%	<0.0001	4
**Post-Traumatic Stress Disorder **	6875	35.9%	20554	29.5%	<0.0001	**14**
**Alcohol use disorder**	5526	28.8%	19650	28.2%	0.0965	1
**Tobacco use disorder**	7644	39.9%	28917	41.5%	<0.0001	3
**Other drug disorder**	4910	25.6%	16340	23.5%	<0.0001	5
**Anxiety**	8269	43.1%	25577	36.7%	<0.0001	**13**
**Depression**	11401	59.5%	35981	51.7%	<0.0001	**16**
**Traumatic Brain Injury**	2217	11.6%	6832	9.8%	<0.0001	6
**Neck pain**	10960	57.2%	34427	49.4%	<0.0001	**16**
**Back Pain**	14645	76.4%	50303	72.3%	<0.0001	**10**
**Cancer**	3911	20.4%	16475	23.7%	<0.0001	8
**Prior opioid Prescription**	16486	86.0%	60589	87.0%	0.0003	3

**Table 3:** Baseline characteristics of the opioid initiator cohort

	Dual Users (*N* = 9032)		Mono Users (VA Only) (*N* = 31000)		*p*-value	ASD (%)
	Mean/*N*	Std/%	Mean/*N*	Std/%		
**Age**	57.5	14.5	61.2	15.0	< 0.0001	**15**
**Gender**					< 0.0001	
**Female**	2246	24.9%	3937	12.7%		**32**
**Male**	6786	75.1%	27,063	87.3%		**32**
**Race**					< 0.0001	
**White American**	3185	35.3%	13,859	44.7%		**19**
**African American**	5185	57.4%	15,198	49.0%		**17**
**Others**	208	2.3%	544	1.8%		4
**Unknown**	454	5.0%	1399	4.5%		2
**Ethnicity**					< 0.0001	
**Non-Hispanics**	8514	94.3%	29,263	94.4%		1
**Hispanics**	291	3.2%	781	2.5%		4
**Unknown**	227	2.5%	956	3.1%		3
**Comorbidity**						
**Hypertension**	5753	63.7%	21,603	69.7%	< 0.0001	**13**
**Diabetes Mellitus**	3066	33.9%	10,895	35.1%	0.0353	3
**Post-Traumatic Stress Disorder**	3041	33.7%	8271	26.7%	< 0.0001	**15**
**Alcohol use disorder**	2259	25.0%	7970	25.7%	0.1804	2
**Tobacco use disorder**	3135	34.7%	11,352	36.6%	0.0009	4
**Other drug disorder**	4910	54.4%	16,340	52.7%	< 0.0001	3
**Anxiety**	3527	39.1%	10,045	32.4%	< 0.0001	**14**
**Depression**	4832	53.5%	14,008	45.2%	< 0.0001	**17**
**Traumatic Brain Injury**	967	10.7%	2705	8.7%	< 0.0001	7
**Neck pain**	4502	49.8%	13,119	42.3%	< 0.0001	**15**
**Back Pain**	6114	67.7%	19,046	61.4%	< 0.0001	**13**
**Cancer**	1590	17.6%	6400	20.6%	< 0.0001	8
**Prior opioid Prescription**	6362	70.4%	21,995	71.0%	0.3450	1

**Fig. 4:**
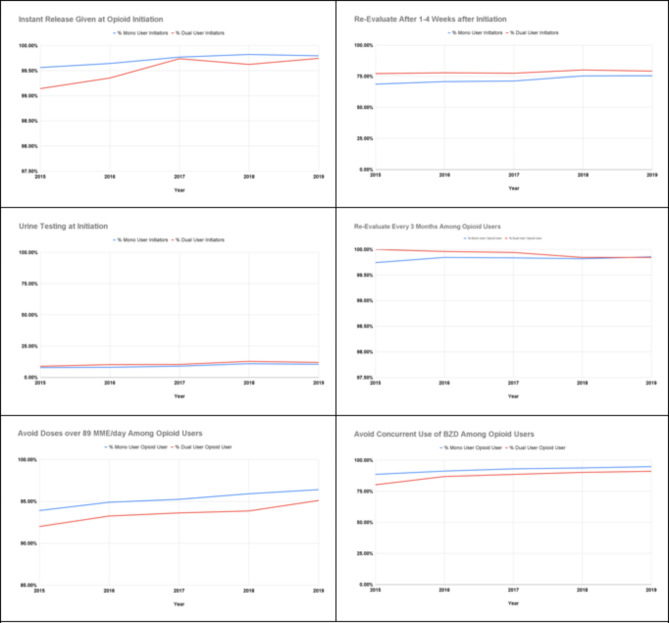
Concordant care rates by year

As shown in Table 4, in both dual and mono users, the rates of care consistent with guidelines were relatively high except for ‘Urine Drug Screening at Initiation’ (11.2% vs. 8.9%). Two outcomes including ‘Initiate Opioids with Immediate Release’ and ‘Re-evaluate Every 3 Months’ had concordant care rates > 99% in both groups, followed by ‘Avoid More than 89 Morphine Milligram Equivalents/Day’ (93.3% vs 95.0%), ‘Avoid Concurrent Prescription of Benzodiazepines’ (88.7% vs 91.5%), and ‘Re-evaluate Between 1–4 Weeks of Initiation’ (78.5% vs 71.5%). The rate changed in parallel in two groups by year as shown in Fig. 4.

**Table 4:** Concordant care in DC and Baltimore VA from 2015-2019

**Opioid Initiators**						
	**Dual-System Users (*N*=9032)**		**Mono Users (*N*=31000)**		***p*-value**	**ASD (%)**
	**N**	**%**	**N**	**%**		
**Initiate opioids with immediate release**	8996	99.6%	30904	99.7%	0.1946	2
**Re-evaluate between 1-4 weeks of initiation**	7092	78.5%	22167	71.5%	<0.0001	**16**
**Urine drug screening at initiation**	1009	11.2%	2763	8.9%	<0.0001	8
**Opioid Users**						
	**Dual-System User (*N*=19164)**		**Mono User ** **(*N*=69621)**		***p*-value**	**ASD (%)**
	**N**	**%**	**N**	**%**		
**Re-evaluate every 3 months**	19146	99.9%	69487	99.8%	0.0035	3
**Avoid more than 89 morphine milligram equivalents/day**	17988	93.3%	66137	95.0%	<0.0001	7
**Avoid concurrent prescription of benzodiazepines**	16995	88.7%	63733	91.5%	<0.0001	9

**Fig. 5:**
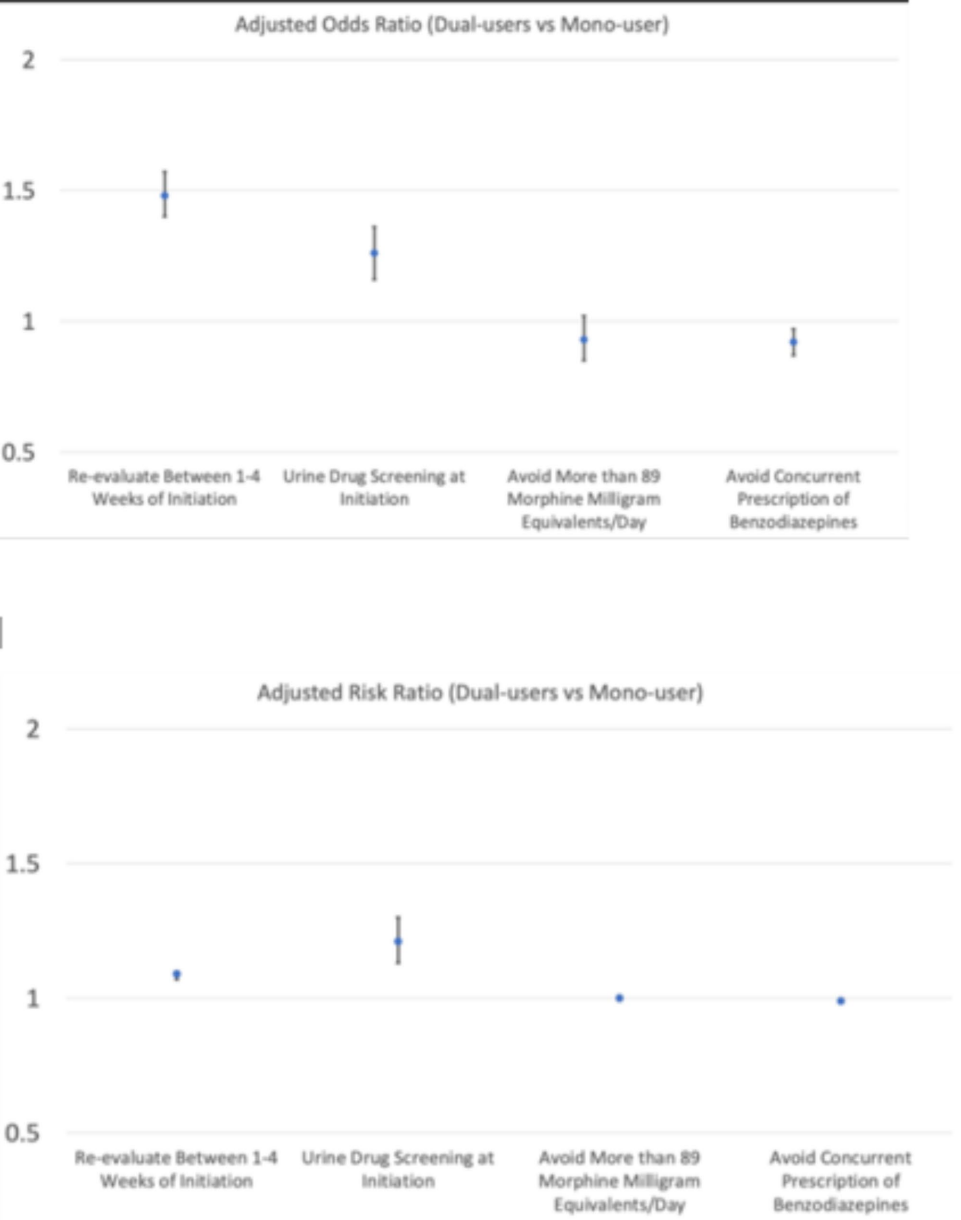
Risk estimates of outcomes (Dual vs mono users)

Compared to the mono users, the dual users were significantly more likely to ‘re-evaluate between 1–4 weeks of initiation’ and have ‘urine drug screening at initiation’ after adjusting for other covariates (Fig. 5). As for the outcomes of ‘avoid more than 89 morphine milligram equivalents/days’, there was no independently significant difference between two group. However, the dual users had significantly lower odds to ‘avoid concurrent prescription of benzodiazepines’ than mono users, but there was no difference when measuring relative risk.

## Discussion

### Significance

In both mono and dual-system users, guideline concordant rates related to opioid therapy are generally high, but there is notable variation on rates depending on the particular guideline. The least commonly observed recommended practice was Urine Drug screening on initiation with only 8.9% of mono-user opioid initiators and 11.2% of dual-user initiators having evidence of having done this within the recommended time frame, or at all. This may be due to its applicability to acute vs. chronic opioid prescriptions, as it was measured by our study. The most commonly followed guideline was ‘initiate opioids with immediate release’ and ‘re-evaluating opioid patients for continued need for an opioid every 3 months’, with as much as 99.6% of mono-users with being treat with opioids having enough visits to justify their treatment-days, and as high as 99.7% of dual-users meeting the same criteria.

There was no consistent pattern of either group of patients outperforming the other in terms of guideline adherence. For example, dual-users had higher rates of re-evaluation between 1 and 4 weeks of initiation and urine screening at initiation, but lower rates of avoiding concurrent benzodiazepine and opioid use. A possible explanation for dual-users having slightly higher rates of concurrent prescriptions, is due to outside clinicians not having access to full prescription records, such as from a patient’s VA care. A slightly clearer trend is that through the course of the study (2015–2019), rates of all guideline adherence increased across all outcomes.

### Implications

Given the findings of our own prior studies [23–25], it may be natural to assume that the rates of guideline concordant care are higher in mono users. Guideline adherence in general is often not ideal [28, 29]. Indeed, more recent versions of the CDC and DoD guidelines have moved away from rigid adherence to specific guidelines such as daily MME limitations, and urine drug screening [9, 20]. However, in the VA system, dual system users received similar or higher rates of guideline concordant care in the context of opioid therapy. For example, nearly all patients receive immediate release formulations as opposed to sustained or extended-release formulations, which is concordant with guidelines. This suggests the cause of higher rates of opioid initiation, use, and misuse involves care outside of VA care.

### Limitations

Guidelines have many recommendations, but some are more difficult to measure than others. We selected several recommendations that are both clinically important and more feasible to measure on the large EHR dataset. Care that is non-concordant with guidelines can still be clinically appropriate, but we cannot assess the individual context within this large set of patients. Several of the outcomes we used are intended to guide long-term opioid use in the treatment of chronic pain, but we did not differentiate between acute and chronic opioid use in our analysis. We opted to proceed with our method of non-differentiation as our goal was not to judge the extent to which clinicians strictly adhered to any particular guideline, but rather to explore differences between cohorts. It is not always known *ex-ante* which patients a clinician treats will end up requiring long-term opioid therapy, and the safety issues addressed by these guidelines remain relevant in both acute and chronic care.

Due to procedural limitations, we were unable to identify the prescribing clinician associated with any given opioid prescription, and thus were unable to identify whether they were VA or CCP clinicians. Therefore, we cannot fully attribute all guideline concordant care for the mixed VA and CCP group to non-VA clinicians.

Our definition of initiation only required that a patient had not taken an opioid within 1 year of their “initiating prescription”. Our ability to determine if a patient had ever been given an opioid is limited by what is available in the VA EHR, and may not include all outside prescriptions, or prescriptions given before the EHR began collecting permanent data. Our definition of initiation may not be exactly the same as each individual prescriber, so their choice to apply the guidelines may not be considered discordant to all observers.

Urine drug screening at initiation appears as a specific recommendation in the 2016 version of the CDC Clinical Practice Guidelines for prescribing opioids. However, initiation as an impetus for urine drug screening is not mentioned in subsequent guidelines from the CDC nor in the VA/DoD guidelines which offer recommendations which are less stringent than other guidelines and may allow room for deviation.

We did not determine whether any specific outpatient encounter addressed chronic pain, or any ongoing opioid prescriptions. Thus, the requirement that the patient had at least 1 visit per 90-days of opioid prescription is a less-than-precise measure of adherence to this guideline. A more nuanced approach would have required using NLP to examine encounter notes, which was outside of the scope of this project.

### Future work

We plan to explore and examine other guideline recommendations relating to risk mitigation such as the prescribing of naloxone to patients deemed at higher risk of overdose. We also plan to study potential coordination issues among dual-user patients as a source of their higher rates of opioid initiation and misuse. Providers and practice settings play important roles in guideline concordant care, which we hope to analyze in future studies.

## Conclusion

We examined adherence to VA/DoD guidelines on opioid prescriptions in a sample of 19,614 dual-System user opioid users, 69,621 mono system opioid users, 9032 dual-system opioid initiators, and 31,000 mono system opioid initiators (Fig. 3). Overall rates of guideline concordant care were high among both dual system initiators and mono system initiators, except when the specific recommendation is less stringent. Guideline concordant care in the VA does not seem to explain the difference in rates of opioid initiation, continued use, and misuse use between mono and dual system users, which have been reported by our team and other researchers.

## Supplementary Information

Supplementary Material 1.

Supplementary Material 2.

## Data Availability

The data utilized in this study were sourced from the VA corporate data warehouse and accessed through The VA Informatics and Computing Infrastructure (VINCI). It is important to note that this data is not publicly available and cannot be shared openly. Researchers seeking access to the data may explore options for collaboration with the relevant research authorities at the VA.
