# Supplementary material for: Guideline concordant opioid therapy in Veterans receiving VA and community care
Source: BMC Health Serv Res. 2024 Oct 26;24:1284. doi: 10.1186/s12913-024-11742-1 (PMC11515256; doi:10.1186/s12913-024-11742-1)
Supplement: Supplementary file 1 — Supplementary Material 1. [file 12913_2024_11742_MOESM1_ESM.docx]

**Appendix I**

**Outcomes, by Cohort Year**

| **Guideline** | **2015** | **2016** | **2017** | **2018** | **2019** |
| --- | --- | --- | --- | --- | --- |
| **Initiate Opioids with Immediate Release** | **9917** | **8863** | **7923** | **6930** | **6267** |
| **% Initiators** | **99.55%** | **99.57%** | **99.76%** | **99.77%** | **99.78%** |
| **Mono-User** | **9570** | **6708** | **5635** | **5071** | **3920** |
| **% Mono-User Initiators** | **99.56%** | **99.64%** | **99.77%** | **99.82%** | **99.80%** |
| **Dual-User** | **347** | **2155** | **2288** | **1859** | **2347** |
| **% Dual-User Initiators** | **99.14%** | **99.35%** | **99.74%** | **99.62%** | **99.75%** |
| **Urine Screening at Initiation** | **780** | **763** | **743** | **795** | **691** |
| **% Initiators** | **7.83%** | **8.57%** | **9.36%** | **11.45%** | **11.00%** |
| **Mono-User** | **749** | **542** | **506** | **556** | **410** |
| **% Mono-User Initiators** | **7.79%** | **8.05%** | **8.96%** | **10.94%** | **10.44%** |
| **Dual-User** | **31** | **221** | **237** | **239** | **281** |
| **% Dual-User Initiators** | **8.86%** | **10.19%** | **10.33%** | **12.81%** | **11.94%** |
| **Re-Evaluate Within 1-4 Weeks of Initiation** | **6869** | **6448** | **5797** | **5319** | **4826** |
| **% Initiators** | **68.95%** | **72.44%** | **72.99%** | **76.58%** | **76.83%** |
| **Mono-User** | **6599** | **4760** | **4020** | **3825** | **2963** |
| **% Mono-User Initiators** | **68.65%** | **70.71%** | **71.18%** | **75.30%** | **75.43%** |
| **Dual-User** | **270** | **1688** | **1777** | **1494** | **1863** |
| **% Dual-User Initiators** | **77.14%** | **77.82%** | **77.46%** | **80.06%** | **79.18%** |
| **Re-Evaluate Every 3 Months** | **22876** | **20334** | **17847** | **14854** | **12722** |
| **% Opioid User** | **99.75%** | **99.87%** | **99.87%** | **99.83%** | **99.85%** |
| **Mono-User** | **22088** | **15348** | **12776** | **11012** | **8263** |
| **% Mono-User Opioid Users** | **99.74%** | **99.84%** | **99.84%** | **99.82%** | **99.85%** |
| **Dual-User** | **788** | **4986** | **5071** | **3842** | **4459** |
| **% Dual-User Opioid Users** | **100.00%** | **99.96%** | **99.94%** | **99.84%** | **99.84%** |
| **Avoid More than 89 MME/day** | **21523** | **19241** | **16941** | **14194** | **12226** |
| **% Opioid User** | **93.85%** | **94.50%** | **94.80%** | **95.39%** | **95.96%** |
| **Mono-User** | **20798** | **14589** | **12190** | **10582** | **7978** |
| **% Mono-User Opioid Users** | **93.92%** | **94.91%** | **95.26%** | **95.92%** | **96.41%** |
| **Dual-User** | **725** | **4652** | **4751** | **3612** | **4248** |
| **% Dual-User Opioid Users** | **92.01%** | **93.26%** | **93.63%** | **93.87%** | **95.12%** |
| **Avoid Concurrent Opioid and BZD Overlap** | **20249** | **18354** | **16398** | **13814** | **11913** |
| **% Opioid User** | **88.30%** | **90.15%** | **91.76%** | **92.84%** | **93.50%** |
| **Mono-User** | **19617** | **14022** | **11904** | **10344** | **7846** |
| **% Mono-User Opioid Users** | **85.54%** | **68.87%** | **66.61%** | **69.52%** | **61.58%** |
| **Dual-User** | **632** | **4332** | **4494** | **3470** | **4067** |
| **% Dual-User Opioid Users** | **80.20%** | **86.85%** | **88.57%** | **90.18%** | **91.07%** |
