# Supplementary material for: Guideline concordant opioid therapy in Veterans receiving VA and community care
Source: BMC Health Serv Res. 2024 Oct 26;24:1284. doi: 10.1186/s12913-024-11742-1 (PMC11515256; doi:10.1186/s12913-024-11742-1)
Supplement: Supplementary file 2 — Supplementary Material 2. [file 12913_2024_11742_MOESM2_ESM.docx]

**Appendix II**

**Odds Ratios**

| **Re-evaluate Between 1-4 Weeks of Initiation** | | | | |
| --- | --- | --- | --- | --- |
|  | Unadjusted OR | | Adjusted OR* | |
|  | OR (95% CI) | p-value | OR (95% CI) | p-value |
| Mixed vs VA Group | 1.45 (1.37 , 1.53) | <.0001 | 1.48 (1.40 , 1.57) | <.0001 |
| Age | 1.01 (1.01 , 1.01) | <.0001 | 1.01 (1.01 , 1.01) | <.0001 |
| Female vs Male | 0.97 (0.91 , 1.03) | 0.3647 | 1.11 (1.04 , 1.19) | 0.0023 |
| African American vs White Americans | 0.87 (0.83 , 0.91) | <.0001 | 0.87 (0.83 , 0.92) | <.0001 |
| Other Race vs White Americans | 0.87 (0.74 , 1.03) | 0.1066 | 0.96 (0.81 , 1.14) | 0.6490 |
| Hispanics vs Non-Hispanics | 0.94 (0.82 , 1.07) | 0.3400 | 1.06 (0.92 , 1.22) | 0.4207 |
| HTN | 1.40 (1.34 , 1.47) | <.0001 | 1.12 (1.06 , 1.19) | 0.0001 |
| DM | 1.43 (1.36 , 1.50) | <.0001 | 1.25 (1.18 , 1.32) | <.0001 |
| PTSD | 1.23 (1.17 , 1.30) | <.0001 | 1.19 (1.12 , 1.26) | <.0001 |
| Alcohol Addiction | 1.02 (0.97 , 1.08) | 0.3806 | 0.93 (0.87 , 1.00) | 0.0399 |
| Tobacco Addition | 1.07 (1.02 , 1.12) | 0.0076 | 1.04 (0.98 , 1.09) | 0.1976 |
| OtherDrugs Addiction | 1.07 (1.01 , 1.13) | 0.0208 | 1.08 (1.00 , 1.16) | 0.0461 |
| Anxiety | 1.15 (1.10 , 1.21) | <.0001 | 1.04 (0.99 , 1.10) | 0.1386 |
| Depression | 1.19 (1.13 , 1.24) | <.0001 | 1.06 (1.01 , 1.12) | 0.0311 |
| TBI | 1.07 (0.98 , 1.15) | 0.1161 | 0.99 (0.91 , 1.08) | 0.8339 |
| Neck pain | 1.22 (1.16 , 1.28) | <.0001 | 1.13 (1.07 , 1.19) | <.0001 |
| Back pain | 1.12 (1.07 , 1.17) | <.0001 | 0.98 (0.94 , 1.04) | 0.5525 |
| Cancer | 1.50 (1.41 , 1.59) | <.0001 | 1.29 (1.21 , 1.37) | <.0001 |
| OpioidsHx | 1.14 (1.09 , 1.20) | <.0001 | 0.90 (0.85 , 0.95) | 0.0002 |
| *OR generated using proc genmod with repeated statement | | | | |

| **Urine Drug Screening at Initiation** | | | | |
| --- | --- | --- | --- | --- |
|  | Unadjusted OR | | Adjusted OR* | |
|  | OR (95% CI) | p-value | OR (95% CI) | p-value |
| Mixed vs VA Group | 1.29 (1.19 , 1.39) | <.0001 | 1.26 (1.16 , 1.36) | <.0001 |
| Age | 0.99 (0.98 , 0.99) | <.0001 | 0.99 (0.98 , 0.99) | <.0001 |
| Female vs Male | 0.82 (0.74 , 0.91) | 0.0001 | 0.8 (0.72 , 0.90) | 0.0001 |
| African American vs White Americans | 1.10 (1.02 , 1.18) | 0.0094 | 0.82 (0.76 , 0.88) | <.0001 |
| Other Race vs White Americans | 0.97 (0.74 , 1.27) | 0.8177 | 0.87 (0.66 , 1.15) | 0.3266 |
| Hispanics vs Non-Hispanics | 0.69 (0.54 , 0.88) | 0.0028 | 0.62 (0.48 , 0.81) | 0.0003 |
| HTN | 0.86 (0.80 , 0.92) | <.0001 | 0.94 (0.86 , 1.03) | 0.2046 |
| DM | 0.83 (0.78 , 0.90) | <.0001 | 0.97 (0.89 , 1.05) | 0.4347 |
| PTSD | 1.63 (1.52 , 1.75) | <.0001 | 1.07 (0.98 , 1.16) | 0.1117 |
| Alcohol Addiction | 2.54 (2.37 , 2.73) | <.0001 | 1.29 (1.17 , 1.41) | <.0001 |
| Tobacco Addition | 1.99 (1.86 , 2.13) | <.0001 | 1.28 (1.18 , 1.38) | <.0001 |
| OtherDrugs Addiction | 3.27 (3.04 , 3.51) | <.0001 | 2.29 (2.08 , 2.52) | <.0001 |
| Anxiety | 1.50 (1.40 , 1.61) | <.0001 | 1.04 (0.96 , 1.13) | 0.3199 |
| Depression | 1.75 (1.63 , 1.88) | <.0001 | 1.12 (1.03 , 1.22) | 0.0072 |
| TBI | 1.61 (1.45 , 1.78) | <.0001 | 1.16 (1.04 , 1.29) | 0.0084 |
| Neck pain | 1.22 (1.14 , 1.31) | <.0001 | 1.01 (0.94 , 1.09) | 0.7287 |
| Back pain | 1.28 (1.19 , 1.37) | <.0001 | 1.10 (1.02 , 1.19) | 0.0195 |
| Cancer | 0.65 (0.59 , 0.71) | <.0001 | 0.79 (0.71 , 0.88) | <.0001 |
| OpioidsHx | 1.19 (1.10 , 1.28) | <.0001 | 0.96 (0.89 , 1.05) | 0.3981 |
| *OR generated using proc genmod with repeated statement | | | | |

| **Avoid More than 89 Morphine Milligram Equivalents/Day** | | | | |
| --- | --- | --- | --- | --- |
|  | Unadjusted OR | | Adjusted OR* | |
|  | OR (95% CI) | p-value | OR (95% CI) | p-value |
| Mixed vs VA Group | 0.95 (0.86 , 1.05) | 0.3405 | 0.93 (0.85 , 1.02) | 0.1179 |
| Age | 1.00 (1.00 , 1.01) | 0.0005 | 1.02 (1.01 , 1.02) | <.0001 |
| Female vs Male | 1.73 (1.50 , 1.99) | <.0001 | 1.71 (1.47 , 1.99) | <.0001 |
| African American vs White Americans | 1.58 (1.45 , 1.72) | <.0001 | 1.63 (1.49 , 1.79) | <.0001 |
| Other Race vs White Americans | 2.51 (1.67 , 3.78) | <.0001 | 2.21 (1.46 , 3.34) | 0.0002 |
| Hispanics vs Non-Hispanics | 1.59 (1.17 , 2.17) | 0.0034 | 1.67 (1.21 , 2.30) | 0.0019 |
| HTN | 0.84 (0.74 , 0.94) | 0.0035 | 0.81 (0.70 , 0.92) | 0.0015 |
| DM | 1.00 (0.90 , 1.11) | 0.9636 | 1.02 (0.92 , 1.13) | 0.6793 |
| PTSD | 1.10 (0.98 , 1.24) | 0.0906 | 1.26 (1.13 , 1.41) | <.0001 |
| Alcohol Addiction | 0.91 (0.81 , 1.02) | 0.1065 | 1.18 (1.03 , 1.35) | 0.0157 |
| Tobacco Addition | 0.65 (0.58 , 0.71) | <.0001 | 0.69 (0.62 , 0.76) | <.0001 |
| OtherDrugs Addiction | 0.76 (0.68 , 0.86) | <.0001 | 0.78 (0.68 , 0.89) | 0.0004 |
| Anxiety | 0.89 (0.80 , 0.99) | 0.0260 | 0.97 (0.87 , 1.08) | 0.5598 |
| Depression | 0.83 (0.75 , 0.91) | 0.0002 | 0.85 (0.76 , 0.95) | 0.0052 |
| TBI | 1.02 (0.87 , 1.21) | 0.7752 | 1.13 (0.97 , 1.31) | 0.1143 |
| Neck pain | 0.94 (0.85 , 1.03) | 0.1915 | 0.98 (0.89 , 1.08) | 0.6915 |
| Back pain | 0.87 (0.78 , 0.97) | 0.0120 | 0.95 (0.85 , 1.05) | 0.3226 |
| Cancer | 0.77 (0.67 , 0.89) | 0.0003 | 0.73 (0.64 , 0.84) | <.0001 |
| OpioidsHx | 0.73 (0.64 , 0.83) | <0.0001 | 0.78 (0.68 , 0.89) | 0.0004 |
| *OR generated using proc genmod with repeated statement | | | | |

| **Avoid Opioid/Benzodiazepine overlap** | | | | |
| --- | --- | --- | --- | --- |
|  | Unadjusted OR | | Adjusted OR* | |
|  | OR (95% CI) | p-value | OR (95% CI) | p-value |
| Mixed vs VA Group | 0.87 (0.82 , 0.92) | <.0001 | 0.92 (0.87 , 0.97) | 0.0041 |
| Age | 1.01 (1.01 , 1.02) | <.0001 | 1.01 (1.01 , 1.01) | <.0001 |
| Female vs Male | 0.60 (0.56 , 0.65) | <.0001 | 0.71 (0.66 , 0.77) | <.0001 |
| African American vs White Americans | 1.67 (1.57 , 1.77) | <.0001 | 1.82 (1.71 , 1.94) | <.0001 |
| Other Race vs White Americans | 1.01 (0.82 , 1.24) | 0.8933 | 1.10 (0.90 , 1.36) | 0.3547 |
| Hispanics vs Non-Hispanics | 0.78 (0.66 , 0.93) | 0.0050 | 1.14 (0.96 , 1.36) | 0.1434 |
| HTN | 1.15 (1.08 , 1.22) | <.0001 | 0.91 (0.84 , 0.99) | 0.0202 |
| DM | 1.15 (1.08 , 1.22) | <.0001 | 1.07 (1.01 , 1.15) | 0.028 |
| PTSD | 0.55 (0.52 , 0.58) | <.0001 | 0.74 (0.69 , 0.79) | <.0001 |
| Alcohol Addiction | 0.95 (0.89 , 1.01) | 0.1095 | 1.15 (1.05 , 1.25) | 0.0015 |
| Tobacco Addition | 0.93 (0.88 , 0.99) | 0.0210 | 1.04 (0.98 , 1.11) | 0.2318 |
| OtherDrugs Addiction | 0.89 (0.83 , 0.95) | 0.0005 | 0.97 (0.88 , 1.06) | 0.4768 |
| Anxiety | 0.42 (0.39 , 0.44) | <.0001 | 0.55 (0.51 , 0.59) | <.0001 |
| Depression | 0.52 (0.49 , 0.55) | <.0001 | 0.77 (0.72 , 0.82) | <.0001 |
| TBI | 0.71 (0.65 , 0.78) | <.0001 | 0.95 (0.87 , 1.04) | 0.2465 |
| Neck pain | 0.73 (0.68 , 0.77) | <.0001 | 0.89 (0.83 , 0.94) | 0.0001 |
| Back pain | 0.78 (0.73 , 0.82) | <.0001 | 0.97 (0.91 , 1.04) | 0.4425 |
| Cancer | 1.04 (0.97 , 1.11) | 0.2684 | 0.95 (0.88 , 1.02) | 0.1564 |
| OpioidsHx | 0.78 (0.72 , 0.84) | <.0001 | 0.89 (0.82 , 0.96) | 0.0038 |
| *OR generated using proc genmod with repeated statement | | | | |

**Risk Ratios**

| **Re-evaluate Between 1-4 Weeks of Initiation** | | | | |
| --- | --- | --- | --- | --- |
|  | Unadjusted RR | | Adjusted RR* | |
|  | RR (95% CI) | p-value | RR (95% CI) | p-value |
| Mixed vs VA Group | 1.10 (1.08 , 1.11) | <.0001 | 1.09 (1.07 , 1.10) | <.0001 |
| Age | 1.00 (1.00 , 1.00) | <.0001 | 1.00 (1.00 , 1.00) | <.0001 |
| Female vs Male | 0.99 (0.98 , 1.01) | 0.3681 | 1.03 (1.01 , 1.05) | 0.0021 |
| African American vs White Americans | 0.96 (0.95 , 0.98) | <.0001 | 0.97 (0.96 , 0.98) | <.0001 |
| Other Race vs White Americans | 0.96 (0.92 , 1.01) | 0.1230 | 1.00 (0.96 , 1.04) | 0.9078 |
| Hispanics vs Non-Hispanics | 0.98 (0.95 , 1.02) | 0.3509 | 1.01 (0.98 , 1.05) | 0.4540 |
| HTN | 1.10 (1.09 , 1.12) | <.0001 | 1.04 (1.02 , 1.06) | <.0001 |
| DM | 1.10 (1.08 , 1.11) | <.0001 | 1.05 (1.04 , 1.07) | <.0001 |
| PTSD | 1.06 (1.04 , 1.07) | <.0001 | 1.04 (1.03 , 1.06) | <.0001 |
| Alcohol Addiction | 1.01 (0.99 , 1.02) | 0.3786 | 0.98 (0.97 , 1.00) | 0.0435 |
| Tobacco Addition | 1.02 (1.00 , 1.03) | 0.0072 | 1.01 (1.00 , 1.02) | 0.1939 |
| OtherDrugs Addiction | 1.02 (1.00 , 1.03) | 0.0190 | 1.02 (1.00 , 1.04) | 0.0334 |
| Anxiety | 1.04 (1.02 , 1.05) | <.0001 | 1.01 (1.00 , 1.03) | 0.0995 |
| Depression | 1.05 (1.03 , 1.06) | <.0001 | 1.01 (1.00 , 1.03) | 0.0853 |
| TBI | 1.02 (1.00 , 1.04) | 0.1093 | 1.00 (0.98 , 1.02) | 0.8471 |
| Neck pain | 1.05 (1.04 , 1.07) | <.0001 | 1.03 (1.02 , 1.04) | <.0001 |
| Back pain | 1.03 (1.02 , 1.04) | <.0001 | 1.00 (0.99 , 1.01) | 0.8556 |
| Cancer | 1.10 (1.09 , 1.12) | <.0001 | 1.05 (1.04 , 1.07) | <.0001 |
| OpioidsHx | 1.04 (1.02 , 1.05) | <.0001 | 0.98 (0.97, 1.00) | 0.0125 |
| *RR generated using proc genmod with repeated statement | | | | |

| **Urine Drug Screening at Initiation** | | | | |
| --- | --- | --- | --- | --- |
|  | Unadjusted RR | | Adjusted RR* | |
|  | RR (95% CI) | p-value | RR (95% CI) | p-value |
| Mixed vs VA Group | 1.26 (1.17 , 1.34) | <.0001 | 1.21 (1.13 , 1.30) | <.0001 |
| Age | 0.99 (0.99 , 0.99) | <.0001 | 0.99 (0.99 , 0.99) | <.0001 |
| Female vs Male | 0.84 (0.76 , 0.92) | 0.0001 | 0.82 (0.74 , 0.91) | <.0001 |
| African American vs White Americans | 1.09 (1.02 , 1.16) | 0.0094 | 0.85 (0.79 , 0.90) | <.0001 |
| Other Race vs White Americans | 0.97 (0.76 , 1.24) | 0.8179 | 0.87 (0.68 , 1.12) | 0.2780 |
| Hispanics vs Non-Hispanics | 0.71 (0.56 , 0.89) | 0.0032 | 0.66 (0.52 , 0.83) | 0.0004 |
| HTN | 0.87 (0.81 , 0.93) | <.0001 | 0.96 (0.89 , 1.04) | 0.2992 |
| DM | 0.85 (0.79 , 0.91) | <.0001 | 0.97 (0.90 , 1.04) | 0.4142 |
| PTSD | 1.55 (1.46 , 1.66) | <.0001 | 1.07 (0.99 , 1.14) | 0.0714 |
| Alcohol Addiction | 2.29 (2.16 , 2.44) | <.0001 | 1.25 (1.15 , 1.35) | <.0001 |
| Tobacco Addition | 1.86 (1.75 , 1.97) | <.0001 | 1.25 (1.17 , 1.34) | <.0001 |
| OtherDrugs Addiction | 2.83 (2.66 , 3.01) | <.0001 | 2.03 (1.87 , 2.21) | <.0001 |
| Anxiety | 1.44 (1.35 , 1.53) | <.0001 | 1.04 (0.97 , 1.11) | 0.2716 |
| Depression | 1.66 (1.56 , 1.77) | <.0001 | 1.12 (1.04 , 1.20) | 0.0040 |
| TBI | 1.52 (1.39 , 1.66) | <.0001 | 1.12 (1.02 , 1.23) | 0.0137 |
| Neck pain | 1.20 (1.13 , 1.27) | <.0001 | 1.01 (0.95 , 1.08) | 0.6885 |
| Back pain | 1.25 (1.17 , 1.33) | <.0001 | 1.08 (1.01 , 1.16) | 0.0271 |
| Cancer | 0.67 (0.62 , 0.73) | <.0001 | 0.81 (0.74 , 0.89) | <.0001 |
| OpioidsHx | 1.17 (1.09 , 1.26) | <.0001 | 0.97 (0.90 , 1.04) | 0.4180 |
| *RR generated using proc genmod with repeated statement | | | | |

| **Avoid MRRe than 89 MRRphine Milligram Equivalents/Day** | | | | |
| --- | --- | --- | --- | --- |
|  | Unadjusted RR | | Adjusted RR* | |
|  | RR (95% CI) | p-value | RR (95% CI) | p-value |
| Mixed vs VA Group | 1.00 (0.99 , 1.00) | 0.3464 | 1.00 (0.99 , 1.01) | 0.1159 |
| Age | 1.00 (1.00 , 1.00) | <.0001 | 1.00 (1.00 , 1.00) | <.0001 |
| Female vs Male | 1.02 (1.02 , 1.02) | <.0001 | 1.02 (1.01 , 1.02) | <.0001 |
| African American vs White Americans | 1.02 (1.02 , 1.02) | <.0001 | 1.02 (1.02 , 1.02) | <.0001 |
| Other or unknown Race vs White Americans | 1.03 (1.02 , 1.04) | <.0001 | 1.02 (1.02 , 1.04) | <.0001 |
| Hispanics vs Non-Hispanics | 1.02 (1.01 , 1.03) | 0.0003 | - | - |
| HTN | 0.99 (0.99 , 1.00) | 0.0024 | - | - |
| DM | 1.00 (1.00 , 1.00) | 0.9638 | 1.00 (1.00 , 1.00) | 0.8052 |
| PTSD | 1.00 (1.00 , 1.01) | 0.0843 | 1.01 (1.00 , 1.01) | 0.0036 |
| Alcohol Addiction | 1.00 (0.99 , 1.00) | 0.1139 | 1.00 (1.00 , 1.01) | 0.1174 |
| Tobacco Addition | 0.98 (0.98 , 0.99) | <.0001 | 0.98 (0.98 , 0.99) | <.0001 |
| OtherDrugs Addiction | 0.99 (0.98 , 0.99) | <.0001 | - | - |
| Anxiety | 0.99 (0.99 , 1.00) | 0.0284 | - | - |
| Depression | 0.99 (0.99 , 1.00) | 0.0002 | 0.99 (0.99 , 1.00) | 0.0007 |
| TBI | 1.00 (0.99 , 1.01) | 0.7730 | - | - |
| Neck pain | 1.00 (0.99 , 1.00) | 0.1923 | - | - |
| Back pain | 0.99 (0.99 , 1.00) | 0.0101 | - | - |
| Cancer | 0.99 (0.98 , 1.00) | 0.0008 | - | - |
| OpioidsHx | 0.99 (0.98 , 0.99) | <.0001 | - | - |
| *RR generated using proc genmod with repeated statement  Some covariates were not included in the model, because the model couldn’t be converged otherwise. | | | | |

| **Avoid Opioid/Benzodiazepine overlap** | | | | |
| --- | --- | --- | --- | --- |
|  | Unadjusted RR | | Adjusted RR* | |
|  | RR (95% CI) | p-value | RR (95% CI) | p-value |
| Mixed vs VA Group | 0.99 (0.98 , 0.99) | <.0001 | 0.99 (0.99 , 1.00) | 0.0007 |
| Age | 1.00 (1.00 , 1.00) | <.0001 | 1.00 (1.00 , 1.00) | <.0001 |
| Female vs Male | 0.95 (0.94 , 0.96) | <.0001 | 0.96 (0.95 , 0.97) | <.0001 |
| African American vs White Americans | 1.04 (1.04 , 1.05) | <.0001 | 1.05 (1.04 , 1.06) | <.0001 |
| Other Race vs White Americans | 1.00 (0.98 , 1.02) | 0.7827 | 1.01 (0.99 , 1.03) | 0.3210 |
| Hispanics vs Non-Hispanics | 0.98 (0.95 , 0.99) | 0.0113 | 1.01 (0.99 , 1.02) | 0.4845 |
| HTN | 1.01 (1.01 , 1.02) | <.0001 | 0.99 (0.99 , 1.00) | 0.0140 |
| DM | 1.01 (1.01 , 1.02) | <.0001 | 1.00 (1.00 , 1.01) | 0.4781 |
| PTSD | 0.94 (0.94 , 0.95) | <.0001 | 0.95 (0.95 , 0.96) | <.0001 |
| Alcohol Addiction | 1.00 (0.99 , 1.00) | 0.1131 | 1.01 (1.00 , 1.01) | 0.0687 |
| Tobacco Addition | 0.99 (0.99 , 1.00) | 0.0218 | 1.00 (1.00 , 1.00) | 0.9999 |
| OtherDrugs Addiction | 0.99 (0.98 , 1.00) | 0.0007 | 0.99 (0.98 , 1.00) | 0.0022 |
| Anxiety | 0.92 (0.92 , 0.93) | <.0001 | - | - |
| Depression | 0.95 (0.94 , 0.95) | <.0001 | - | - |
| TBI | 0.97 (0.96 , 0.98) | <.0001 | - | - |
| Neck pain | 0.97 (0.97 , 0.98) | <.0001 | - | - |
| Back pain | 0.98 (0.97 , 0.98) | <.0001 | - | - |
| Cancer | 1.00 (1.00 , 1.00) | 0.2637 | 0.99 (0.99 , 1.00) | 0.0355 |
| OpioidsHx | 0.98 (0.98 , 0.99) | <.0001 | - | - |
| *RR generated using proc genmod with repeated statement  Some covariates were not included in the model, because the model couldn’t be converged otherwise. | | | | |
